# Effects of Casirivimab/Imdevimab Monoclonal Antibody Treatment among Vaccinated Patients Infected by SARS-CoV-2 Delta Variant

**DOI:** 10.3390/v14030650

**Published:** 2022-03-21

**Authors:** Gaetano Cicchitto, Lorena Cardillo, Claudio de Martinis, Paola Sabatini, Rosita Marchitiello, Giovanna Abate, Adele Rovetti, Antonietta Cavallera, Camillo Apuzzo, Francesco Ferrigno, Giovanna Fusco

**Affiliations:** 1 Department of Pneumology, COVID-19 Hospital “M. Scarlato”, 84018 Scafati, Salerno, Italy; gaeint@libero.it (G.C.); francescoferrigno@libero.it (F.F.); 2 Istituto Zooprofilattico Sperimentale del Mezzogiorno, 80055 Portici, Naples, Italy; claudio.demartinis@izsmportici.it (C.d.M.); giovanna.fusco@izsmportici.it (G.F.); 3 Unit of Virology and Microbiology, “Umberto I” Hospital, 84014 Nocera Inferiore, Salerno, Italy; paola.sabatini1@gmail.com; 4 Unit of Clinical Pathology Laboratory, COVID-19 Hospital “M. Scarlato”, 84018 Scafati, Salerno, Italy; rositamarchitiello@libero.it (R.M.); abategiovanna.am@gmail.com (G.A.); adele.rovetti@libero.it (A.R.); 5 Department of Radiology, COVID-19 Hospital “M. Scarlato”, 84018 Scafati, Salerno, Italy; antocava123@gmail.com (A.C.); apuzzorx@gmail.com (C.A.)

**Keywords:** SARS-CoV-2, monoclonal antibody treatment, casirivimab/imdevimab, variants of concern, B.1.617.2, delta variant

## Abstract

There is a growing interest in using monoclonal antibodies (mAbs) in the early stages of Severe Acute Respiratory Syndrome Coronavirus 2 (SARS-CoV-2) infection to prevent disease progression. Little is known about the efficacy of mAbs against the delta variant of concern and its clinical presentations. We evaluated the effect of casirivimab/imdevimab treatment among five delta vaccine breakthrough patients. Symptomatic non-hospitalized vaccinated patients were submitted to nasopharyngeal swabs for the detection of SARS-CoV-2 and Next-Generation Sequencing (NGS). Blood analysis and chest Computed Tomography were also performed. A cocktail of casirivimab/imdevimab was administrated, and patients were monitored weekly. Clinical evolution was evaluated by the regression of the symptoms, negative results by real-time RT-PCR, and by the need of hospitalization: these aspects were considered as significant outcomes. In four cases, symptom reversion and viral load reduction were observed within 2 days and 7 days after mAbs treatment, respectively. Only one case, suffering from thymoma, was hospitalized 2 days later because of respiratory failure, which reverted within 18 days. mAbs treatment seems to be safe and effective against the delta variant and its clinical manifestations.

## 1. Introduction

Since it first appearance, Severe Acute Respiratory Syndrome Coronavirus 2 (SARS-CoV-2), the etiologic agent of Coronavirus Disease 2019 (COVID-19), has created high concern for its morbidity and mortality. Mutations of this novel coronavirus have been observed since the very early stages of the pandemic, and some mutants have emerged and dominantly spread [1]. SARS-CoV-2 is a positive single-stranded RNA virus that shows a moderate nucleotide substitution rate, caused by the error-prone nature of the RNA Polymerase RNA-Dependent (RdRp), that can lead to a rapid viral evolution [2]. However, while some mutations do not have a direct and significant impact on the virus, such as D614G, the first identified mutation, others can provide some characteristics that can improve the survival, such as higher transmissibility, pathogenicity, with induction of a more severe form of disease, or the ability to escape the immunity acquired following natural infections or vaccinations [3]. When these characteristics are reported, we have to deal with Variants of Concern (VOCs) [4]. The B.1.617.2 (delta) VOC was first identified in late 2020 and has become the predominant lineage worldwide, causing in September 2021 99.8% of the COVID-19 cases in Europe [5] and, from August to October 2021, 99.5% of the cases in Italy [6]. This variant has been observed to cause immune-escape, showing six-fold lower sensitivity to serum-neutralizing antibodies from recovered individuals and eight-fold lower sensitivity to vaccine-elicited antibodies compared with wild type [7]. Indeed, delta VOC has proven to cause higher rates of hospitalization than previous ones, even in vaccinated people [5], thus requiring urgent intervention to prevent more severe diseases and heavy pressure on Intensive Care Units (ICUs). The use of monoclonal antibodies (mAbs) arose interest since the beginning of the pandemic based on the evidence of the efficacy of passive immunization during previous Coronavirus epidemics caused by SARS-CoV-1 and Middle East Respiratory Syndrome (MERS)-CoV, that were associated with reduction in both virus replication and mortality due to the antibodies neutralizing activity [8,9,10]. Neutralizing SARS-CoV-2 mAbs are developed against the Spike (S) protein, in order to block viral attachment, host cell entry, and infectivity [11]. More than 100 different mAbs for SARS-CoV-2 have been registered worldwide [12], and currently, three anti-SARS-CoV-2 mAbs have received the emergency use authorization by the Food and Drug Administration (FDA), Italian Medicines Agency (AIFA), bamlanivimab plus etesevimab, casirivimab plus imdevimab, and sotrovimab, all targeting the Receptor Binding Domain (RBD) of the S glycoprotein [13,14], but the emergence of SARS-CoV-2 VOCs, which have demonstrated immune escape both to vaccines and to previous natural infections [3], has created questions on the efficacy of the treatment among these variants. In particular, it has been reported that while the B.1.1.7 (alpha) variant is not refractory to the association of casirivimab plus imdevimab (REGN-CoV2), the B.1.351 (beta) and P.1 (gamma) variants have been found to be resistant to the neutralization activity of the two both casirivimab and imdevimab separately, but their association showed therapeutic efficacy [15]. Moreover, patients infected by the P.1 variant showed high risk of disease progression following bamlanivimab/etesevimab treatment [16]. Little is known about the efficacy of the casirivimab/imdevimab cocktail on the delta variant. The aim of this study was to retrospectively evaluate the outcome of the disease among vaccinated patients infected by B.1.617.2 VOC.

## 2. Materials and Methods

### 2.1. Subjects and Clinical Data

In September–October 2021, five vaccinated patients with two-dose mRNA vaccines were considered as eligible to the administration of mAbs as they showed mild to moderate COVID-19, had symptom onset within 7 days, were non-hospitalized and aged over 12 years, weighed at least 40 kg, did not require supplement oxygen therapy, and were identified to be at increased risk of progression to severe COVID-19, as defined by AIFA for the optimal use of anti-COVID-19 monoclonal antibodies [17]. In order to analyze the various effects of the infection, we used a standardized scale of radiological descriptions [18,19] and criteria suggested in the literature to assess dysregulated systemic inflammation [20,21,22]; symptom burden reported by the patients on questionnaires was classified based on recommendations by the Center for Disease Control and Prevention (CDC) already used in previous works [23,24]. Patients were submitted to nasopharyngeal swab for SARS-CoV-2 molecular examinations (see below), High-Resolution Computer Tomography (HRCT) to obtain the Total Severity Score (TSS). In particular, each of the five lung lobes was assessed for percentage of the lobar involvement and classified as none (0%), minimal (1–25%), mild (26–50%), moderate (51–75%), or severe (76–100%), with corresponded score as 0, 1, 2, 3, or 4. The TSS was reached by summing the five lobe scores (range from 0 to 20) [18]. Next, evaluation of hemoglobin oxygen saturation (SaO_2_), blood analysis, including interleukin 6 (IL-6), C-reactive protein (CRP), D-Dimer, and ferritin, as benchmarks of the inflammation, were performed. Criteria for efficacy were the change from baseline in the SARS-CoV-2 viral load within 10 days after the therapy, the change in symptoms’ burden, as reported by the patients on a questionnaire, and clinical outcomes, which are defined as COVID-19-related hospitalization or access to the emergency room.

### 2.2. Monoclonal Antibodies Administration

All the patients received a cocktail of casirivimab (1200 mg) and imdevimab (1200 mg) (Regeneron/Roche, Roche Registration GmbH, Grenzach-Wyhlen, Germany) by intravenous (IV) infusion according to the U.S. Food and Drug Administration (FDA) and Italian Medicines Agency (AIFA) recommendations [17,25]. After mAbs administration, patients were discharged and weekly ambulatory monitored as outpatients for 30 days, up to the achievement of symptom regression and negative results by real-time RT-PCR. Safety criteria were based on the report of adverse events, such as 72 h post-infusion-related reactions, changes in vital signs or other circumstances, based on the Common Terminology Criteria for Adverse Events (CTCAE) [26].

### 2.3. Nucleic Acids Extraction and Molecular Analysis

The nasopharyngeal swabs of patients 1, 2, and 3 were transferred to the Unit of Virology and Microbiology of “Umberto I” Hospital in Universal Viral Transport Medium (UTM) (Copan, Brescia, Italy). Samples were submitted to multiplex real-time RT-PCR using Allplex SARS-CoV-2 Variants II Assay (Seegene, Seoul, South Korea) for the detection of K417N, K417T, L452R and W152C mutation sites in the Spike (S) gene for the identification of the most reported SARS-CoV-2 VOCs, following the manufacturer’s instructions. Patient 4 and 5 swabs were processed at the Istituto Zooprofilattico Sperimentale del Mezzogiorno (IZSM). Nucleic acids extraction was conducted in Biosafety Level 3 (BLS-3) laboratories. Aliquots of 200 µL of UTM were collected to perform extraction and purification using KingFisher Flex (Thermo Fisher Scientific, Waltham, MA, US) with MVP_2Wash_200_Flex program following manufacturer’s instructions, eluted in a 50 µL final volume and stored at –80 °C until use. Notably, SARS-CoV-2 RT-qPCR was performed in BLS-2 as already described [27].

### 2.4. SARS-CoV-2 Next-Generation Sequencing

Patient 5 tested positive, and the sample was firstly submitted to reverse transcription with AmpliSeq cDNA Synthesis (Illumina) following the manufacturer’s instructions. Next, the cDNA was amplified with AmpliSeqLibrary PLUS for Illumina kit (Illumina Inc., San Diego, CA, USA) and SARS-CoV-2 Research Custom RNA Panel (Illumina). Library preparation was obtained by uniquely indexing the amplification products with AmpliSeq UD Indexes for Illumina, followed by sample library amplification, as per the manufacturer’s protocol. Then, libraries were cleaned up with AMPure XP beads (Beckman Coulter Inc., Brea, CA, USA) and freshly prepared 70% ethanol and diluted in 27 µL of Low TE buffer. Finally, libraries were quantified by using 2 µL of the amplification products with a Qubit dsDNA High-Sensitivity assay kit on Qubit 2.0 instrument (Life Technologies, Eugene, OR, USA), and 1 µL was used to analyze fragment sizes with D1000 screen tape and reagents (Agilent Technologies Inc. Santa Clara, CA, USA) on capillary electrophoresis (Tapestation 2200, Agilent Technologies) (the protocol is available at https://support.illumina.com/content/dam/illumina-support/documents/documentation/chemistry_documentation/ampliseq-for-illumina/ampliseq-for-illumina-custom-and-community-panels-reference-guide-1000000036408-09.pdf, accessed on 12 March 2021).

The sample library was normalized to 2 nM concentration, and a 10 µL pool of normalized libraries was obtained and submitted to denaturation with 10 µL of 0.2 N sodium hydroxide for 5 min at room temperature; then, 10 µL of 200 Mm Tris-HCl pH 7 was added. A final concentration of 20 pM was sequenced on Illumina MiSeq platform (Illumina), using a MiSeqMicro Reagent Kit v2 (300-cycles) (Illumina).

After quality control, FASTQ files were imported and aligned using the Geneious R9 software package (Biomatter). Next, the consensus sequence was obtained and submitted to the GISAID database (http://www.gisaid.org, accessed on 5 November 2021) (Gisaid accession n. EPI_ISL_5934813).

## 3. Results

### 3.1. Patient 1

A 42-year-old smoker man, with a history of secondary immunodeficiency for systemic rheumatic disease, was admitted to the hospital after three days of fever, dry cough, headache, nausea, vomiting, and increasing arthromyalgia. His SaO_2_ was 98%. Real-time RT-PCR showed a cycle threshold (Ct) value of 27. The patient had previously received two doses of COVID-19 vaccine BNT162b2 (Pfizer-BioNTech), which were administered 77 days apart. Inflammation indicators revealed increased values of interleukin 6 (IL-6) and C-reactive protein (CRP), 61.2 pg/L and 51.9 mg/L, respectively, while no signs of interstitial pneumonia or other pulmonary alterations were observed at HRCT. Next, an intravenous infusion of casirivimab/imdevimab was administrated, and symptoms reverted within 2 days. Further molecular examinations revealed a decreased viral load (Ct 34) at day 7, and negative results were obtained 21 days after the administration of the mAbs cocktail.

### 3.2. Patient 2

An 89-year-old non-smoker woman, with a history of Hodgkin lymphoma, systemic hypertension, and diabetes, was found to be SARS-CoV-2 positive by RT-PCR (Ct 15). She had been reporting fever, mild dyspnea, and arthralgia for four days. Inflammatory biomarkers showed increased values of IL-6 (69.2 pg/L) and CRP (107.4 mg/L); HRCT pointed out bilateral interstitial pneumonia, obtaining a TSS of 10/20 [18], even though SaO_2_ was constantly 98%. She had previously received two doses of COVID-19 mRNA1273 vaccine (Spikevax), and the last one was administrated 7 months before the onset of the symptoms; nevertheless, SARS-CoV-2 IgG anti-spike antibodies showed a weak or absent immune response to the vaccination (0.4 U/mL; negative < 0.8 U/mL). She received the mAb cocktail of casirivimab/imdevimab and was discharged. Symptoms disappeared within 2 days, and the patient tested negative for SARS-CoV-2, 19 days following the mAbs treatment.

### 3.3. Patient 3

A 42-year-old non-smoker man, suffering from thymoma, after seven days of fever, dry cough, dyspnea, headache, abdominal disturbances, and fatigue, was found to be SARS-CoV-2 positive (Ct 19). The blood tests showed increased CRP (62.3 mg/L), ferritin (1566 ng/mL), IL6 (72.2 pg/L), and D-dimer (3595 ng/mL), suggesting severe inflammation. HRCT confirmed the presence of bilateral interstitial pneumonia (TSS: 16/20) [18], and SaO_2_ was 92%. He had previously received two doses of BNT162b2 vaccine, which were administered 4 months apart, but the SARS-CoV-2 IgG anti-spike was considered as negative, with results < 0.8 U/mL, thus suggesting a weak or absent immune response to the vaccine or its fading. On the same day of the diagnosis, he received the casirivimab/imdevimab intravenous infusion (1200 mg + 1200 mg). After 48 h, the patient was hospitalized for respiratory failure. He was treated by supportive therapy and oxygen administration, resulting in clinical improvement and regression of respiratory failure in 18 days. Negative results for SARS-CoV-2 by RT-PCR were obtained 29 days after the mAbs treatment.

### 3.4. Patient 4

A 55-year-old non-smoker obese woman (Body Mass Index (BMI): 39), with a history of systemic hypertension and venous thromboembolism, tested positive for SARS-CoV-2 with a Ct value of 18. The woman showed low grade fever, dry cough, headache, myalgia, and fatigue, and her SaO_2_ was 97%. Either HRCT or blood analysis did not reveal signs of hyperinflammation (low-grade alterations of CRP, D-Dimer, and IL6). She had previously received two doses of BNT162b2 vaccine, the second injection was 3 months before the onset of symptoms, and her IgG anti-spike antibodies concentration was 5000 U/mL. She received the mAbs intravenous infusion at the same concentration of the previous cases, after three days from the onset of the illness. Symptoms reverted within 2 days; moreover, a reduction in viral load (Ct 25) was observed at day 7 and there were negative results at day 15 after the treatment.

### 3.5. Patient 5

A 24-year-old smoker obese woman, with a BMI 37.1, the daughter of patient 4, tested positive for SARS-CoV-2 (Ct 25) on a nasopharyngeal swab submitted to NGS that revealed the AY.3 delta VOC. She showed low-grade fever, headache, and fatigue, with a SaO_2_ value of 98%. Neither signs of pulmonary involvement nor blood analysis alterations were observed. She had been previously vaccinated using two doses of BNT162b2, and the second inoculation was administrated 3 months earlier. mAbs were received after three days from the onset of symptoms, which reverted within 2 days. Negative results were obtained by real-time RT-PCR 7 days after the monoclonal antibodies treatment (results are summarized in Table 1).

## 4. Discussion

Since its recognition as a cause of COVID-19, SARS-CoV-2 continues to bring new challenges. In addition, continuous genetic mutations of SARS-CoV-2, occurring during replication of its genome, represent a further demanding threat, caused by the rapid spread of new variants [28], and others might probably appear [29]. Current therapeutic strategies are mainly supportive, which implies that efforts should be directed toward the prevention of disease progression. Due to the lack of a therapy recognized as effective, the “TTT (test–track–treat) strategy” was proposed with the purpose of preventing an exponential surge of COVID-19 cases [30,31,32]. In this context, early treatment of the disease becomes essential. The mAbs are recombinant proteins able to target the S glycoprotein of SARS-CoV-2, preventing it from binding to its cognate receptor ACE2 on host cells; this “neutralizing” effect could inhibit the evolution of the disease to more severe manifestations. At the beginning of 2021, the Food and Drug Administration (FDA), the European Medicines Agency (EMA), and subsequently the Italian Medicine Agency (AIFA) approved an emergency use authorization of some mAbs for COVID-19 non-hospitalized patients (outpatients) with risk factors for worsening, within 7 days after symptoms’ onset, based on the results of published studies [24,33].

The cases we described lead us to several considerations. Therapy with casirivimab/imdevimab seems to be safely and usefully performed on delta variant vaccine breakthrough patients across a range of clinical manifestations; remarkably, the patients were in different phases of the disease, as shown by the radiological and biohumoral data. In all these cases, a favorable effect of the mAbs therapy was observed by a decrease in the viral load in 10 days, and there was an absence of the viral load by Nucleic Acid Amplification Test (NAAT) on nasopharyngeal swab by the 30th day after the therapy. Moreover, no evident adverse effect was observed. Nevertheless, patient 3 developed respiratory failure 48 h after the administration of mAbs. It is described that the use of monoclonal antibodies can cause enhanced respiratory disease (ERD) and antibody-dependent enhancement (ADE) [34], with increased body temperature and oximetry reduction, but, as observed by Yoshida et al., two-dose vaccination shows a two-fold lower risk of oximetry reduction [35]. Thus, it is not possible to distinguish between the occurrence of respiratory failure caused by an adverse event to the administration mAbs and the worsening of COVID-19 [35]. Furthermore, the hospitalization of patient 3 did not lead to invasive or non-invasive mechanical ventilation, and the patient was discharged without oxygen supply; this patient was given the suggested best supportive therapy (including antiviral and corticosteroids), so we can only speculate that mAbs therapy may have contributed to slowing down a further progression of the disease and possibly avoiding the need for mechanical ventilation and access to ICU. Additionally, a recent report highlighted a reduced 28-day mortality among hospitalized patients treated with casirivimab and imdevimab, who were seronegative at baseline [36].

The promptness of intervention seems to be of utmost importance: indeed, the different outcomes between the two patients with pneumonitis could also have been affected by the time lapse between the onset of symptoms and therapy (4 vs. 7 days). Patients 4 and 5, a mother and her daughter, were treated with mAbs; despite this, their SARS-CoV-2 IgG anti-spike protein were strongly positive, as the association of casirivimab/imdevimab was effective across several patients, including SARS-CoV-2 serum antibody-positive at baseline [37]. Indeed, according to the guidelines for the use of mAbs, the evaluation of IgG anti-Spike antibodies is not required. No side effects of the therapy were recorded, and the symptoms regressed quickly. This points out that the mAbs treatment may have had an effect on the patients’ clinical course. Finally, since the spread of SARS-CoV-2 VOC could lead to a limitation on the effectiveness of the vaccination and containment programs, the changing scenario makes it important to better clarify the therapeutic potential of mAbs. Little is known about the efficacy of bamlanivimab/etesevimab and casirivimab/imdevimab, which were two mAbs used against delta VOC: the dominant variant in that period. A recent report highlighted a quick resolution of the symptoms in two delta variant vaccine breakthrough subjects treated with bamlanivimab/etesevimab [38]; our cases present further data in this field, underlining the efficacy of casirivimab/imdevimab against the delta variant in the context of patients with several clinical manifestations.

## 5. Conclusions

In a significant proportion of COVID-19 outpatients, interstitial pneumonia could be observed without signs of respiratory failure; thus, it is possible that mAbs treatment can be effective even in this scenario. Indeed, passive immunization with mAbs could be a therapeutic option to prevent severe manifestations of COVID-19, in particular in vulnerable patients. In this context, the casirivimab/imdevimab seems to be helpful in improving the outcome of COVID-19 patients, with or without interstitial pneumonia.

## Figures and Tables

**Table 1:** Patient analyses and clinical data.

	Age (y)	Sex	Comorbidities	Last Vaccination	SARS-CoV-2 (Ct)	Symptoms	IgG Anti-spike Antibodies (U/mL)	SaO_2_ (%)	IL-6 (pg/L)	CRP (mg/L)	HRCT TSS	Symptom Reversion (Days)
Patient 1	42	M	Systemic Rheumatic disease	2.5 months	27	Fever, dry cough, headache, nausea, vomiting, arthromyalgia	<0.8	98	61.2	51.9	0/20	2
Patient 2	89	F	Hodgkin Lymphoma Hypertension Diabetes	7 months	15	Fever, mild dyspnea, arthralgia	<0.8	98	69.2	107.4	10/20	2
Patient 3	42	M	Thymoma	4 months	19	Fever, dry cough, dyspnea, abdominal disturbances, headache, fatigue	<0.8	92	72.2	62.3	16/20	18
Patient 4	55	F	Obesity, Hypertension, thromboembolism	3 months	18	Fever, dry cough, headache, myalgia, fatigue	5000	97	5.55	7.6	0/20	2
Patient 5	24	F	Obesity	3 months	25	Fever, headache, fatigue	1325	98	4.9	4.1	0/20	2

Ct: cycle threshold; SaO_2_: hemoglobin oxygen saturation; IL-6: interleukin 6; CRP: C-reactive protein; HRCT TSS: high-resolution computer tomography total severity score.

## Data Availability

Not applicable.
